# Immunometabolic Remodeling in Osteosarcopenia: Inflammaging, Mitochondrial Dysfunction, Gut-Derived Metabolites and Therapeutic Opportunities

**DOI:** 10.3390/metabo16080556

**Published:** 2026-08-06

**Authors:** Yichi Zhang, Yuntao Li, Xun Luo, Qingmei Wang, Luwen Zhu, Yan Wang

**Affiliations:** 1 Graduate School, Heilongjiang University of Chinese Medicine, Harbin 150040, China; 2 School of Psychiatry, Wenzhou Medical University, Wenzhou 325035, China; 3 Stroke Biological Recovery Laboratory, Spaulding Rehabilitation Hospital, The Teaching Affiliate of Harvard Medical School, Boston, MA 02129, USA; 4 The Second Affiliated Hospital of Heilongjiang University of Chinese Medicine, Harbin 150001, China

**Keywords:** osteosarcopenia, sarcopenia, osteoporosis, immunometabolism, inflammaging, gut microbiota

## Abstract

Osteosarcopenia—defined as the coexistence of sarcopenia and osteoporosis—is increasingly recognized as a clinically important geriatric syndrome associated with falls, fractures, frailty, disability, and mortality. Beyond the simple coexistence of bone and muscle loss, emerging data suggest that osteosarcopenia may reflect systemic dysregulation of the bone–muscle–immune–metabolic network. In this narrative review, we synthesize evidence linking inflammaging, immune-cell polarization, mitochondrial dysfunction, nutrient metabolic dyshomeostasis, and gut-derived metabolites to the pathogenesis of osteosarcopenia. Multiple pathological processes—including chronic low-grade inflammation, Th17/Treg imbalance, macrophage polarization, oxidative stress, impaired mitophagy, insulin resistance, ectopic fat accumulation, and altered microbial metabolites—may converge to disrupt bone–muscle crosstalk. Notably, direct evidence from osteosarcopenic populations remains limited, and many mechanistic insights are extrapolated from osteoporosis, sarcopenia, and aging models. We further discuss current and emerging therapeutic strategies, including exercise, nutritional interventions, anti-osteoporotic agents, metabolic modulators, mitochondrial-targeted therapies, and gut-directed approaches. Longitudinal cohorts, multi-omics studies, and randomized controlled trials are urgently required to validate immunometabolic biomarkers and develop integrated interventions for osteosarcopenia.

## 1. Introduction

Osteosarcopenia (OS) is commonly defined as the coexistence of sarcopenia (SP) and osteoporosis (OP), characterized by impaired muscle strength and/or performance together with reduced bone mass and deteriorated bone quality [1], imposing a substantial burden on both patients’ families and healthcare systems. With accelerating global population ageing, OS prevalence continues to rise, ranging from approximately 5% to 37% among community-dwelling elderly individuals [2]. A major obstacle in OS research is the absence of a universally accepted diagnostic definition. Different combinations of low bone mineral density (BMD), OP, low appendicular lean mass, reduced grip strength and impaired physical performance have been used, leading to substantial heterogeneity in prevalence estimates and mechanistic studies.

Spatially adjacent muscle and bone maintain close interactive dialogue, exchanging mechanical signals to coordinate bone density and muscle mass while secreting biochemical factors to regulate each other [3]. Muscle functions as an endocrine organ, secreting multiple myokines—such as irisin, interleukins, and insulin-like growth factor 1 (IGF-1)—which directly or indirectly influence bone metabolism. Conversely, bone tissue secretes osteokines, including osteocalcin and sclerostin, to regulate muscle metabolism and function [4]. The musculoskeletal systems are intrinsically interconnected, forming a bidirectional regulatory relationship. Their co-regulatory mechanisms have emerged as a current research focus, yet deeper underlying pathways remain incompletely elucidated. Identifying synergistic intervention targets to achieve co-management of musculoskeletal comorbidities is therefore urgently required.

Immunometabolism represents an emerging field linking cellular metabolism with immune function. Metabolic pathways not only fuel immune cell activation but also direct their differentiation and effector functions in health and disease [5]. In OS, immune–metabolic dysregulation may drive concurrent muscle and bone loss. While mechanisms are partially understood—chronic low-grade inflammation promotes muscle catabolism and impairs regeneration [6]; mitochondrial metabolism is associated with apoptosis in skeletal muscle cells [7]; and gut microbiota dysbiosis contributes to muscle decline [8]—integrated research on immunometabolic regulation of OS remains scarce. This review critically examines key immunometabolic mechanisms in OS, including chronic inflammation, immune cell dysregulation, mitochondrial dysfunction, metabolic disorder, and gut microbiota alterations, to provide novel foundations for OS prevention and treatment.

## 2. Evolution of Diagnostic Definitions and Their Implications

The diagnostic framework for OS has evolved from the separate conceptualisation of muscle wasting and bone loss toward an integrated skeletal–muscular syndrome. In 2018, the European Working Group on Sarcopenia in Older People 2 (EWGSOP2) established low muscle strength (grip strength < 27 kg in men and <16 kg in women) as the primary diagnostic criterion, with low appendicular skeletal muscle mass (<20 kg in men and <15 kg in women) serving as confirmatory evidence [9]. The Asian Working Group for Sarcopenia (AWGS) 2025 consensus update represents a paradigm shift from conceptualising SP as a discrete disease toward promoting muscle health across the life course. For the first time, the diagnostic scope encompasses middle-aged adults (50–64 years), with validated thresholds derived from Asian cohorts. The diagnostic algorithm is streamlined to concurrent low muscle mass and low muscle strength; physical performance is repositioned as an outcome measure, and disease severity grading has been eliminated [10]. For OP, the International Osteoporosis Foundation (IOF) and European Society for Clinical and Economic Aspects of Osteoporosis, Osteoarthritis and Musculoskeletal Diseases (ESCEO) have advocated for the inclusion of trabecular bone score and fracture risk assessment to capture bone quality beyond BMD [11].

The choice of diagnostic algorithm carries profound implications for mechanistic interpretation. Cohorts defined by low lean mass, for instance, differ substantially from those identified by grip strength or gait speed thresholds. Consequently, reported prevalence rates vary considerably across studies, and mechanistic findings may diverge depending on whether the index population is characterised primarily by low muscle mass, impaired muscle function, frailty, metabolic dysregulation, or systemic inflammation. Moreover, the modality used to assess body composition—bioelectrical impedance analysis (BIA), dual-energy X-ray absorptiometry (DXA), or computed tomography (CT)—introduces further variability. These methodological divergences imply that “OS” in one study may represent a partially distinct clinical entity from that in another, hampering the identification of universal biomarkers or therapeutic targets. To advance the field, future studies should explicitly report the diagnostic criteria and sex-specific thresholds employed, and conduct sensitivity analyses comparing alternative definitions. Such standardisation is essential to improve inter-study comparability.

## 3. Literature Search Strategy

We searched PubMed and CNKI databases from inception to March 2026 using combinations of the following terms: “OS,” “SP,” “OP,” “immunometabolism,” “inflammaging,” “mitochondrial dysfunction,” “gut microbiota,” “short-chain fatty acids (SCFAs),” “bile acids,” “metabolomics,” and “immune cells.” Inclusion criteria were original studies, reviews, and meta-analyses related to musculoskeletal aging, bone-muscle interactions, and immunometabolic regulation. Exclusion criteria were conference abstracts and studies lacking mechanistic or clinical relevance. After screening titles and abstracts, 90 relevant references were ultimately cited in this review.

Throughout this review, we distinguish three levels of evidence. Direct evidence refers to studies conducted in individuals explicitly diagnosed with OS. Indirect clinical evidence refers to studies in OP, SP, frailty, diabetes, obesity, or aging cohorts that include bone or muscle outcomes relevant to OS. Mechanistic evidence refers to cell and animal studies that clarify biological pathways but have not yet been validated in OS populations. This distinction provides a clear hierarchy of biological reasoning.

## 4. Immune Mechanisms in OS

### 4.1. Chronic Low-Grade Inflammation

Chronic low-grade inflammation (inflammaging) plays a pivotal role in OS. Alleviating inflammation mitigates damage to muscle and bone, thereby improving the symptoms of OS [12]. Inflammatory responses activate immune cells within muscle and bone, triggering microstructural alterations and tissue damage that drive OS [13]. Pro-inflammatory cytokines—including interleukin (IL)-6 and tumour necrosis factor-α (TNF-α)—disrupt musculoskeletal metabolism and inhibit myogenic and osteogenic processes [6,12]. Conversely, anti-inflammatory cytokines such as IL-10 and transforming growth factor-β (TGF-β) suppress chronic inflammation, enhance regenerative pathways, and maintain musculoskeletal homeostasis [12]. However, inflammaging should not be interpreted solely as increased circulating cytokine concentrations. Within the broader geroscience framework, it is closely connected to cellular senescence, senescence-associated secretory phenotype (SASP) signaling, epigenetic remodeling, and immunosenescence. Inflammaging-associated epigenetic changes, including altered DNA methylation, histone modification, and chromatin accessibility, may reprogram immune and progenitor cells toward a persistent pro-inflammatory state.

Cellular senescence represents a stable cell-cycle arrest induced by telomere erosion, DNA damage, oxidative stress, and oncogene activation. Senescent cells accumulate in aging bone and muscle tissues, where they adopt a pro-inflammatory secretory profile termed the SASP [14]. The SASP encompasses a broad repertoire of cytokines (IL-6, IL-8, TNF-α), chemokines, growth factors, and matrix metalloproteinases that propagate senescence to neighboring cells and remodel the extracellular matrix. Pro-inflammatory cytokines reinforce local inflammation, promote osteoclastogenesis, suppress osteoblast differentiation, impair satellite-cell activation, and favor fibrotic or adipogenic remodeling of musculoskeletal tissue. IL-6, a pleiotropic cytokine secreted by activated monocytes, T cells, and endothelial cells, exerts context-dependent effects. TNF-α, primarily secreted by activated macrophages, is a hallmark of systemic inflammation in ageing. Within bone, TNF-α and IL-6 stimulate osteoblast receptor activator of nuclear factor-κB ligand (RANKL) expression, promoting osteoclast differentiation and bone resorption [15]. IL-6 also mediates estrogen deficiency-induced bone loss [16]. In muscle, IL-6 activates the janus activated kinase (JAK)/signal transducer and activator of transcription (STAT) pathway, upregulating suppressor of cytokine signaling (SOCS) to degrade insulin receptor substrate-1 (IRS1) and accelerate skeletal muscle protein breakdown [17]. TNF-α inhibits the Akt (Protein Kinase B)/mammalian target of rapamycin (mTOR) pathway, impairing myogenesis while stimulating muscle protein degradation via the ubiquitin-proteasome system [18]. Animal studies demonstrate that infusion of inflammatory mediators (e.g., TNF-α, IL-6) induces skeletal muscle protein breakdown and atrophy in rats [19,20]. Anti-inflammatory factors such as IL-10 and TGF-β promote musculoskeletal homeostasis. IL-10 mitigates inflammation by suppressing macrophage activation and pro-inflammatory cytokine release (IL-6, TNF-α, IL-1β), while promoting muscle regeneration through polarization of macrophages from the M1 to M2 phenotype. In IL-10 homozygous knockout mice, IL-6 levels significantly increase by 50 weeks, accompanied by declines in skeletal muscle mass, strength, and function during aging [21]. TGF-β suppresses inflammatory cell activation and proliferation, reducing inflammatory severity. It also promotes muscle stem cell (MuSC) proliferation and differentiation, facilitating myocyte regeneration, and regulates osteoprogenitor cell differentiation and function via Smad signaling pathway activation [12]. Direct evidence in OS remains limited, but indirect evidence from OP, SP, frailty, and aging cohorts consistently links elevated inflammatory markers and senescence-associated pathways to reduced BMD, impaired muscle strength, and poor physical performance.

### 4.2. Immune Cell Dysregulation and Polarization

Immune cells are key regulators of musculoskeletal metabolism. Various immune cell types—including T lymphocyte subsets (TH1, TH2, TH17, regulatory T cells (Tregs)), B lymphocytes, macrophages, dendritic cells, neutrophils, mast cells, and eosinophils—participate in the pathogenesis of OS through direct and indirect mechanisms [22]. Immunosenescence refers to the progressive decline in immune function with aging, characterized by thymic involution, reduced naive T-cell output, expansion of memory and exhausted T-cell pools, and impaired antigen presentation. Concurrently, innate immune cells exhibit heightened basal activation and blunted responses to novel pathogens—a state of “inflammaging” driven by cell-autonomous defects and chronic antigenic stimulation [23]. In the musculoskeletal system, immunosenescence manifests as skewed T-cell receptor repertoires, reduced Treg suppressive function, and increased circulating pro-inflammatory cytokines. Aged macrophages exhibit impaired phagocytosis and exaggerated inflammasome activation, contributing to unresolved inflammation in bone and muscle niches. Epigenetic remodeling constitutes a fundamental mechanism underlying both cellular senescence and immunosenescence. Age-associated alterations in DNA methylation (the “epigenetic clock”), histone modifications, and chromatin accessibility reprogram immune and stromal cells toward pro-inflammatory, catabolic phenotypes [24].

T lymphocyte subsets play a crucial role in musculoskeletal homeostasis. Th17 cells are major producers of IL-17, which promotes osteoclast differentiation by upregulating RANK expression in osteoclast precursors [25] and mediates bone loss via the IL-17 receptor A (IL-17RA) and adaptor protein Nuclear Factor Kappa-B activator 1 (Act1) [26]. Enhanced glycolysis promotes rapid proliferation of Th1 and Th17 cells and secretion of effector cytokines (IL-17, Interferon-gamma (IFN-γ)), whereas glycolytic inhibition impairs Th1 function. Hypoxia-inducible factor-1α (HIF-1α) binds the IL-17 promoter, promoting Th17 differentiation and glycolytic capacity, thereby driving Th17 proliferation, survival, and effector function [27]. Tregs exhibiting anti-inflammatory properties, suppress osteoclast generation through cell-contact-dependent mechanisms and secrete cytokines such as IL-10 and TGF-β to inhibit osteoclast differentiation, thereby protecting bone [22]. Tregs also release amphiregulin, a growth factor that stimulates MuSC activity and facilitates muscle regeneration [28]. Tregs rely primarily on fatty acid oxidation (FAO) and oxidative phosphorylation (OXPHOS) for energy, enabling sustained suppressive function. In OS age- and inflammation-driven metabolic shifts toward glycolysis may promote Th17 expansion over Tregs, disrupting musculoskeletal homeostasis.

B cells mediate humoral immunity via antibody secretion and regulate inflammatory responses and immune homeostasis through cytokine production. In inflammatory environments, B cells promote bone resorption while inhibiting bone formation by altering the RANKL/osteoprotegerin (OPG) ratio [29]. Animal studies show that B lymphocyte-deficient mice exhibit OPG deficiency and develop OP more frequently than controls [30]. In B-cell acute lymphoblastic leukemia, excessive RANKL expression increases osteoclasts, causes trabecular bone loss, destroys the epiphyseal growth plate, and reduces adipocyte mass [31]. Beyond conventional B cells, regulatory B cells (Bregs) also modulate bone homeostasis. Bregs produce cytokines such as TGF-β1, IL-10, and IL-35 that regulate osteogenic differentiation. Notably, IL-35 drives Breg differentiation via the STAT1/STAT3 signaling pathway [32]. Glucose metabolism is crucial for B cell function. Upon activation, B cells increase glucose uptake, yet carbon tracing shows glucose is diverted from glycolysis to the pentose phosphate pathway (PPP), nucleotide synthesis, and fatty acid synthesis. While tenfold glucose reduction does not impair proliferation or differentiation, it compromises antibody class switching [33]. Activated B cells exhibit enhanced tricarboxylic acid (TCA) cycle and OXPHOS activity, but glucose is not the primary energy source. Glutamine drives B cell differentiation, proliferation, and class switching, contributing to DNA replication and biomass accumulation, and at least partially fuels heightened TCA cycle activity [34].

Macrophages are vital immune system components characterized by remarkable plasticity and dynamism. Through secretion of diverse bioactive molecules, they mediate inflammation initiation, maintenance, and resolution—a process termed macrophage polarization [35]. Macrophage polarization can be categorized into the classically activated M1 phenotype and the alternatively activated M2 phenotype. Under pro-inflammatory conditions, M0 macrophages polarize to the M1 phenotype, shifting metabolism toward anaerobic glycolysis. This pathway rapidly generates ATP and precursors for pro-inflammatory mediators (TNF-α, IL-1β, IL-6) [36]. Pyruvate dehydrogenase kinase 1 (PDK1) promotes M1 polarization via HIF-1α-mediated glycolytic reprogramming, intensifying pro-inflammatory responses [37]. Resulting M1 macrophages promote osteoclast precursor proliferation and differentiation, inhibit muscle regeneration, and accelerate muscle atrophy. The acidic microenvironment formed by lactate, a glycolytic byproduct, further enhances osteoclast activity and bone resorption. Animal studies show that M1 infiltration exacerbates muscle/bone atrophy following peripheral nerve injury; suppression of M1 macrophages at the injury site prevents muscle and bone atrophy [38]. To prevent chronic inflammation following aberrant M1 activation, M0 and M1 macrophages can polarize to anti-inflammatory M2 phenotype under stimulation by Th2 cytokines and immune complexes [39]. This process involves a metabolic shift from glycolysis to the TCA cycle and OXPHOS [40], typically mediated by AMP-activated protein kinase (AMPK) activation. M2 macrophages are the predominant phenotype in healthy tissues, playing a pivotal role in anti-inflammatory processes. They mitigate muscle atrophy, promote muscle regeneration, and restore skeletal homeostasis [41]. Their secreted factors (IL-10, TGF-β, vascular endothelial growth factor (VEGF)) downregulate osteoclast-associated genes (RANKL) and upregulate osteoblast-associated genes (Runt-related transcription factor 2 (Runx2)), thereby inhibiting bone resorption and promoting bone formation [42,43]. Therefore, rationally regulating M1/M2 balance may suppress chronic inflammation and improve musculoskeletal metabolic function in OS patients. Table 1 illustrates the dual effects of key cytokines on the skeletal and muscular systems in OS.

Immune regulation in OS is best understood as a dynamic multicellular network rather than the isolated action of individual immune-cell populations. Macrophages, T cells, B cells, osteocytes, osteoblasts, osteoclasts, satellite cells, fibro-adipogenic progenitors, and mesenchymal stem cells communicate through cytokines, chemokines, extracellular vesicles, metabolic substrates, and cell-contact-dependent signals. In bone, osteocytes sense mechanical and metabolic stress and regulate osteoblast and osteoclast activity through RANKL, OPG, sclerostin, and inflammatory mediators. In muscle, satellite cells and fibro-adipogenic progenitors require coordinated immune-cell transitions from early pro-inflammatory responses to later pro-regenerative signals.

## 5. Metabolic Perturbations in OS

### 5.1. Mitochondrial Dysfunction and Oxidative Stress

Mitochondria are the core organelles of cellular energy metabolism. The musculoskeletal system, characterized by high energy demands and susceptibility to hypoxic–ischemic injury, is particularly vulnerable to energy depletion resulting from mitochondrial dysfunction. As a central mechanism of cellular aging, mitochondrial dysfunction plays a pivotal role in the onset and progression of OS. Mitochondrial dysfunction affects the musculoskeletal system through three primary mechanisms: accumulation of mitochondrial DNA (mtDNA) mutations, excessive reactive oxygen species (ROS) production, and impaired autophagy [44].

With advancing age, mtDNA mutations accumulate in somatic cells [45], leading to diminished metabolic efficiency and excessive ROS generation. ROS accumulation induces oxidative stress, damaging osteocytes and disrupting bone microarchitecture. Dobson et al. [46] reported that mice with mtDNA mutations exhibited more pronounced bone loss, reduced bone formation rates, decreased osteoblast numbers, and elevated osteoclast counts compared with age-matched controls. Additionally, mtDNA mutations disrupt genomic stability, exacerbating pro-inflammatory cytokine accumulation and fostering a chronic inflammatory microenvironment. This inhibits osteoblast differentiation while enhancing osteoclast activity, disrupting the balance between bone formation and resorption and accelerating age-related bone loss [47].

With aging, mitochondria generate excessive ROS. Gradual ROS accumulation exceeding antioxidant capacity leads to oxidative stress [48], which damages mitochondrial function and creates a vicious cycle. In muscle tissue, oxidative stress impairs regeneration by suppressing satellite cell proliferation and differentiation, activating the nuclear factor-κB (NF-κB) signaling pathway, and accelerating muscle protein degradation [49]. In osteoblasts, oxidative stress inhibits osteogenic activity, disrupts bone matrix formation and mineralization, enhances osteoclast activity, and exacerbates OP [50]. Feng et al. [51] reported that inhibiting ROS production restores mitochondrial function and suppresses osteoclast activity.

Mitophagy serves as a pivotal mechanism for maintaining mitochondrial homeostasis. Insufficient autophagy leads to accumulation of damaged mitochondria and increased ROS, causing cellular damage [52] conversely, excessive autophagy may fragment healthy mitochondria and trigger cell death [53]. In skeletal muscle, PINK1/Parkin-mediated mitophagy is essential for MuSC differentiation [54], and autophagy-related gene 7 (ATG7) deficiency leads to severe muscular atrophy and neuromuscular junction deterioration [55]. In bone tissue, aging-associated advanced glycation end-products (AGEs) accelerate bone marrow mesenchymal stem cell (BMSC) senescence by suppressing mitophagy, thereby impairing osteogenic differentiation [44]. Sirtuin 3 (Sirt3) reverses AGE-induced dysfunction by restoring mitophagy, maintaining the balance between osteogenic and adipogenic differentiation [44]. These findings suggest that targeted modulation of mitophagy may represent a promising therapeutic strategy for preventing and treating OS syndrome.

However, mitochondrial health is maintained not solely by mitophagy but by an integrated quality control network encompassing mitochondrial biogenesis, dynamics (fusion and fission), the mitochondrial unfolded protein response (UPRmt), and mitochondrial-derived peptides (MDPs).

Mitochondrial biogenesis, orchestrated by peroxisome proliferator-activated receptor-γ coactivator-1α (PGC-1α) and nuclear respiratory factors (NRF1/2), declines with aging in both skeletal muscle and bone. Reduced PGC-1α expression impairs OXPHOS capacity, decreases ATP production, and increases ROS generation [56]. In muscle, a decrease in the mRNA and protein levels of PGC-1α, PGC-1β and TFAM leads to impaired mitochondrial biosynthesis (the PGC-1α-NRF1-TFAM pathway), resulting in disrupted mitochondrial quality control and a reduction in muscle mass [57]. In bone, PGC-1α activation in osteoblasts enhances differentiation and mineralization, while its suppression shifts MSC fate toward adipogenesis [58].

Mitochondrial dynamics—the balance between fusion and fission—regulate organelle morphology, function, and quality control. Fusion facilitates complementation of damaged mitochondrial components and sustains OXPHOS efficiency, whereas fission segregates damaged segments for mitophagic clearance [59]. In aging muscle, excessive fission fragments the mitochondrial network and reduces respiratory capacity. In osteoblasts, DRP1-mediated mitochondrial fission is necessary for differentiation, but chronic overactivation induces apoptosis [60]. The succinate-SUCNR1 axis promotes mitochondrial fission via the extracellular signal-regulated kinase 1/2 (ERK1/2)/DRP1 signaling pathway, sustaining a vicious cycle of mitochondrial dysfunction and inflammation in the bone microenvironment [61].

UPRmt is a stress-activated transcriptional program that restores proteostasis by upregulating mitochondrial chaperones (e.g., mtHsp70, Hsp60) and proteases (e.g., ClpP, LONP1) [62]. Experimental evidence indicates that activation of UPRmt transcription factors can restore mitochondrial proteostasis and delay muscle aging [63], although its specific role in OS remains to be elucidated.

MDPs, including humanin and MOTS-c, represent a novel class of hormetic signaling molecules encoded by mitochondrial open reading frames. Humanin is detectable in bone and muscle, and emerging evidence indicates a role in skeletal disease through the regulation of osteoclasts and osteoblasts. It may protect against bone disorders by suppressing osteoclastogenesis via activation of AMPK [64]. MOTS-c translocates to the nucleus and modulates metabolic gene expression, enhancing glucose uptake in skeletal muscle and improving insulin sensitivity. In aged mice, MOTS-c administration improves muscle insulin signaling and physical performance [65].

### 5.2. Dysregulation of Glucose Metabolism

Abnormal glucose metabolism is closely associated with OS, promoting chronic low-grade inflammation, mitochondrial dysfunction, and abnormal lipid metabolism, thereby contributing to disease progression [66]. Type 2 diabetes mellitus (T2DM), the most prevalent form of glucose metabolism disorder, significantly impacts musculoskeletal metabolism through multiple mechanisms. Hyperglycemia disrupts AKT and GSK-3β—key regulators of glucose and bone metabolism—thereby impairing osteoblast differentiation and exacerbating bone metabolic imbalance [67,68,69]. Concurrently, hyperglycemia promotes AGE accumulation, which induces skeletal muscle oxidative stress via ROS generation and activates the PERK/FOXO1 pathway, exacerbating muscle atrophy [70]. Furthermore, AGEs bind to receptor for advanced glycosylation end-products (RAGE), activating downstream NF-κB signaling to increase inflammatory factor release, thereby inhibiting osteoblast activity and enhancing osteoclast differentiation [71,72]. Glucose-lowering agents show therapeutic potential for musculoskeletal disorders. For instance, metformin improves insulin resistance while promoting osteoblast differentiation and inhibiting osteoclast activity, thereby supporting bone health [73]. Thorough investigation of glucose metabolism abnormalities in OS is therefore essential for clinical management.

### 5.3. Dysregulation of Lipid Metabolism and Fat Infiltration

Lipid metabolism correlates with oxidative stress. Fat infiltration increases pro-inflammatory factors, triggering inflammation and impairing muscle function, whereas lipid catabolism plays a crucial role in osteogenic differentiation. Altered lipid metabolism is prominent in the elderly, individuals with type 2 diabetes, and obese populations [74]. In skeletal muscle, fat infiltration serves as a hallmark of muscle loss and aging. 1-Palmitoyl glycerophosphocholine and pentadecanoate are both associated with lipid metabolism; the former correlates with increased muscle mass, while the latter correlates with decreased muscle mass [74]. Polyunsaturated fatty acids such as α-linolenic acid and eicosapentaenoic acid show therapeutic potential for SP due to their anti-inflammatory properties [75]. Age-related lipid metabolism abnormalities, coupled with chronic inflammation, lead to fat redistribution to visceral or skeletal muscle, resulting in reduced muscle strength and function [76]. Markers reflecting dysregulation of glucose and lipid metabolism, including the triglyceride-glucose index (TyG index) and plasma atherogenic index (AIP), positively correlate with SP risk, with this association partially mediated by inflammation and oxidative stress [77]. During osteogenesis, matrix production and mineralization demand substantial energy, which can be supplied by lipid catabolism [78,79]. Free fatty acids (FFAs) released by bone marrow adipocytes are taken up by BMSCs, undergo mitochondrial β-oxidation, and participate in OXPHOS, providing energy for osteogenic differentiation [80]. This suggests that key enzymes in FAO may play a positive role in regulating osteoblast metabolism.

### 5.4. Dysregulation of Amino Acid Metabolism

Amino acid metabolic balance is crucial for maintaining musculoskeletal quality, influencing muscle and bone metabolism through protein synthesis, degradation, and signaling pathways. Recent Mendelian randomization studies reveal an association between glycine and muscle strength decline [74]. Beyond protein synthesis, glycine exerts immunomodulatory and cytoprotective effects and is essential for skeletal muscle regeneration [81]. Glycine also inhibits neurotransmitter release, regulating inter-neuronal signaling—a fundamental mechanism for controlling skeletal muscle activity [82]. Arginine promotes osteogenesis and inhibits adipocyte formation via Wnt5a and NFATc signaling, suggesting positive effects on bone metabolism [83].

Branched-chain amino acids (BCAAs)—leucine, isoleucine, and valine—serve as critical regulators of muscle protein turnover. Leucine activates mTOR complex 1 (mTORC1) signaling, stimulating muscle protein synthesis and inhibiting autophagy [84]. However, BCAA catabolism is impaired in insulin-resistant states, leading to accumulation of branched-chain α-keto acids (BCKAs) that exacerbate oxidative stress [85].

Methionine metabolism and one-carbon metabolism are intimately linked to epigenetic regulation and redox homeostasis. Methionine restriction extends lifespan in model organisms, partly by altering S-adenosylmethionine (SAM)-dependent DNA and histone methylation [86]. Research indicates that methionine inhibits the differentiation of osteoblast precursors in culture, consistent with the established role of methionine metabolism in maintaining pluripotency and directing lineage commitment in stem cells. Conversely, methionine supplementation in fully differentiated osteoblasts suppresses osteoblast markers, including the phenotype-critical transcription factors ATF4 and RUNX2 [87].

One-carbon metabolism, supported by serine and glycine, provides methyl groups for epigenetic enzymes and nucleotide precursors for DNA repair. L-Serine maintains one-carbon unit availability by entering the folate and methionine cycles; these units are utilised in diverse metabolic pathways, including purine and pyrimidine synthesis, as well as in the methylation of proteins and nucleic acids via SAM. Serine serves as the principal one-carbon donor, being converted to glycine and N^5^,N^10^-methylenetetrahydrofolate via serine hydroxymethyltransferase (SHMT) [88].

Glutamine is the most abundant non-essential amino acid in the circulation and serves multiple metabolic and physiological functions, including nitrogen transport, acid–base balance maintenance, and energy provision for immune cells and intestinal epithelial cells. In skeletal muscle, glutamine regulates the balance between protein synthesis and breakdown. Plasma glutamine levels in athletes with overtraining syndrome remain persistently below baseline, and glutamine supplementation alleviates exercise-induced muscle damage in professional athletes [89]. In bone, glutamine metabolism regulates bone marrow stem cell proliferation, lineage commitment, and osteoblast differentiation [90]. Furthermore, it replenishes TCA cycle intermediates via α-ketoglutarate and supports redox homeostasis by providing precursors for glutathione synthesis, thereby enhancing cellular tolerance to oxidative stress. Enhanced glutamine metabolism ensures osteoblast survival and functional stability by providing energy and sustaining the antioxidant system. The interplay between glutamine metabolism and immune cell function in the OS microenvironment warrants further investigation, particularly given the high glutamine demands of both proliferating osteoblasts and infiltrating immune cells. Future OS studies should integrate targeted amino acid profiling with dietary assessment, inflammatory markers, muscle function, and bone outcomes to clarify whether amino acid signatures can identify treatable metabolic subtypes. Sex differences in amino acid metabolism regulating musculoskeletal homeostasis likely reflect hormonal variation; the mechanisms underlying these differences in OS patients require further elucidation.

### 5.5. Gut Microbiota as a Metabolic-Immune Mediator

The gut microbiota—a complex ecosystem of bacteria, archaea, viruses, and fungi [91]—serves as a pivotal metabolic-immune hub in the pathogenesis of OS. Gut dysbiosis contributes to immune–metabolic disorders in OS. Studies indicate that OS patients exhibit microbial alterations, including Eggerthella enrichment and Lachnospiraceae and Blautia depletion, alongside potential upregulation of purine, pyrimidine, cysteine, and methionine metabolism, suggesting metabolic dysregulation 8.

Central to this interaction are SCFAs—primarily acetate, propionate, and butyrate [92]. Beyond their role as energy substrates, SCFAs function as signaling ligands for specific metabolic sensors, most notably the G protein-coupled receptors GPR41 (FFAR3) and GPR43 (FFAR2) [93]. Acetate interacts with GPR43 to inhibit histone deacetylase (HDAC), thereby suppressing inflammation. Propionate activates GPR43 and suppresses HDAC, promoting regulatory Treg proliferation and function. Butyrate acts on GPR43 and GPR109A, inhibits HDAC, and activates the NLRP3 inflammasome to promote IL-18 expression. Its capacity to induce Foxp3+ Tregs and IL-10-producing T cells further enhances anti-inflammatory properties [94].

SCFAs enhance intestinal barrier integrity by modulating host immunity (e.g., promoting Treg differentiation) and suppressing pro-inflammatory T cells [91]. They also improve intestinal epithelial absorption capacity, enhancing calcium and phosphorus uptake [95]. Additionally, SCFAs stimulate IGF-1 release and regulate gut hormones, thereby promoting skeletal health. Studies confirm that microbiota-derived SCFAs serve as key mediators in the gut-bone axis, regulating both osteoblasts and osteoclasts [96]. Skeletal muscle cells can utilize SCFAs as an energy source 95. These metabolites may also activate regulatory pathways (UCP2-AMPK-ACC, PGC1-α), increasing ATP production and enhancing muscle fiber metabolic efficiency [97]. Although these findings support a role for SCFAs in musculoskeletal homeostasis, direct evidence in osteosarcopenic patients remains scarce. Most studies have been conducted in OP, SP or metabolic disease models. Moreover, inter-individual variation in diet, medication exposure, frailty status and sequencing methodology limits cross-study comparability. Therefore, whether SCFA supplementation can prevent or reverse OS requires validation in well-designed randomized trials.

Bile acids are end products of cholesterol metabolism, facilitating digestion and absorption of dietary lipids and fat-soluble vitamins. The gut microbiota–bile acid–farnesoid X receptor (FXR)-FGF15/19 signaling pathway is a key component of the gut–skeletal muscle axis [98]. In the intestine, primary conjugated bile acids are deconjugated by microbes with bile salt hydrolase (BSH) activity and metabolized by distal gut microbiota into secondary bile acids [99]. Studies indicate that bile acids activate the nuclear receptor FXR, inducing expression of fibroblast growth factor 15 (FGF15) in mice and its human homolog FGF19 (collectively termed FGF15/19) [100]. Through enterohepatic circulation, FGF15/19 reaches the liver and binds to the fibroblast growth factor receptor 4/β-klotho (FGFR4/KLB) complex, thereby regulating bile acid synthesis. Intestinal FXR expression correlates with skeletal muscle mass, grip strength, and microbial diversity, suggesting that age-related OS may involve gut microbiota dysbiosis and subsequent FXR-FGF15/19 signaling disruption [101]. Guo et al. demonstrated that FGF19 protects skeletal muscle from obesity-induced atrophy by suppressing muscle wasting marker expression and enhancing myogenic differentiation-related molecule expression [102].

Beyond SCFAs and bile acids, additional microbial metabolites are emerging as relevant regulators of immune function and skeletal muscle biology. Indole derivatives, produced by tryptophan-metabolising bacteria, activate the aryl hydrocarbon receptor (AhR) on immune cells and maintain intestinal barrier integrity. Indole-3-aldehyde, in particular, exhibits anti-inflammatory properties in chronic inflammatory diseases, downregulating IL-1β, IL-6, and TNF-α expression [103]. Trimethylamine N-oxide (TMAO), a dietary choline metabolite produced by gut microbiota, stimulates osteoclast differentiation and inflammation through activation of the NF-κB signalling pathway in vitro. These findings suggest that elevated circulating TMAO may contribute to bone loss and fracture risk in older adults [104]. Urolithin A, a polyphenolic compound produced by gut microbiota through the metabolism of ellagic acid and ellagitannins, promotes mitochondrial health by inducing mitophagy in preclinical ageing models and older adults. Long-term urolithin A supplementation significantly enhances skeletal muscle endurance and improves metabolic markers of mitochondrial function in older adults [105]. Polyamines (putrescine, spermidine, and spermine) are synthesised by both host tissues and gut microbiota, and regulate autophagy, cell proliferation, and differentiation. Polyamine supplementation enhances autophagic flux in muscle cells, thereby preserving muscle function [106].

**Figure 1:**
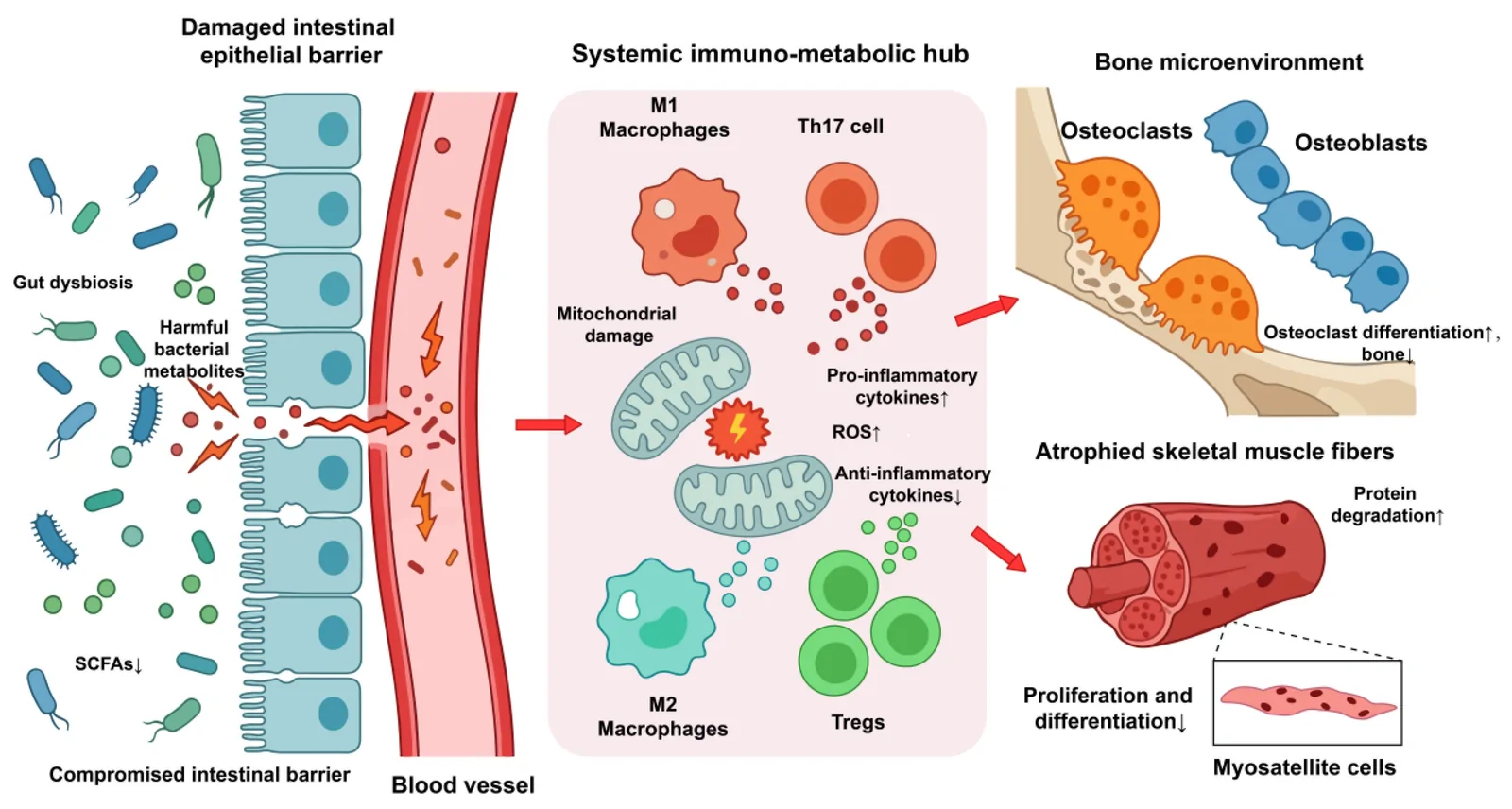
The role of immunometabolism in the pathogenesis of OS.

Supplementation with specific probiotics, such as Bifidobacterium and Lactobacillus, can regulate intestinal microbial balance and enhance barrier function [107], thereby potentially alleviating disease symptoms. Animal studies demonstrate that microbiota-derived metabolites influence skeletal muscle protein turnover, contributing to increased muscle mass and strength [108]. Clinical studies indicate that probiotic therapy in elderly patients may improve BMD and muscle function [109]. Future research should focus on precision interventions using single probiotic strains. Figure 1 illustrates the role of immunometabolism in the pathogenesis of OS.

## 6. Synergistic Crosstalk: The Immune–Metabolic Axis in OS

The immune–metabolic microenvironment is pivotal in determining the progression of OS. Rather than functioning in isolation, immune cells and metabolic pathways converge at specific “interaction nodes,” where the local metabolic microenvironment directly dictates immune cell phenotypes, and conversely, immune polarization reshapes local tissue and systemic metabolism. Gut microbiota metabolites, particularly SCFAs, exert crucial regulatory roles in this interplay by modulating immune cell activity and metabolic pathways. In-depth investigation of these synergistic mechanisms may provide novel directions for prevention and treatment strategies.

### 6.1. Metabolic Control of Immune-Cell Polarization

The metabolic state of the musculoskeletal microenvironment acts as a primary switch for immune cell polarization and effector function. Under hypoxic conditions, inhibition of the respiratory chain triggers the pathological accumulation of succinate [110]. This elevation suppresses dioxygenase activity through product inhibition and stabilizes HIF-1α, which subsequently drives the secretion of pro-inflammatory cytokines (e.g., IL-1β) and the production of ROS, ultimately impairing the osteogenic differentiation of mesenchymal stem cells (MSCs) [61]. Simultaneously, PDK1 facilitates M1 macrophage polarization via HIF-1α-mediated glycolytic reprogramming, amplifying the inflammatory milieu. These M1 macrophages transfer oxidatively damaged mitochondria to MSCs through microvesicles, inducing cellular injury and metabolic remodeling. Such metabolic disruption further accumulates succinate within MSCs by inhibiting succinate dehydrogenase (SDH) activity. Furthermore, the succinate-SUCNR1 axis promotes mitochondrial fission via the ERK1/2/dynamin-related protein 1 (DRP1) signaling pathway. This persistent mitochondrial dysfunction sustains a vicious cycle of succinate accumulation, inflammation, and ROS production. These ROS, acting as secondary messengers, subsequently induce osteoclastogenesis and exacerbate bone resorption [61].

### 6.2. Local Metabolite Accumulation and Musculoskeletal Degeneration

Systemic metabolic disorders—particularly dysregulation of glucose and lipid metabolism—profoundly reshape the local immune landscape. For instance, chronic hyperglycaemia promotes the accumulation of AGEs, which, upon binding to their receptor (RAGE), activate downstream NF-κB signalling. This activation triggers robust secretion of pro-inflammatory cytokines while concurrently suppressing osteoblast function, enhancing osteoclastogenesis, and inducing muscle protein catabolism. In parallel, age-related ectopic fat infiltration within skeletal muscle exacerbates local inflammation by recruiting and activating pro-inflammatory immune cells. Collectively, these interconnected metabolic disturbances drive macrophage polarization toward the glycolytic M1 phenotype. This impairs muscle regenerative capacity while amplifying osteoclast-mediated bone resorption, thereby accelerating the progression of OS.

### 6.3. Gut-Derived Metabolites as Systemic Immunometabolic Mediators

Gut microbiota-derived SCFAs serve as both an energy source and pivotal immunometabolic regulators of systemic immune homeostasis. SCFAs bind to specific immune cell receptors, including free fatty acid receptors (FFARs) and inhibit HDACs, thereby modulating immune cell differentiation, activation, and function. SCFAs modulate macrophage, neutrophil, and dendritic cell activity. Butyrate, in particular, inhibits macrophage production of pro-inflammatory cytokines and promotes differentiation toward an anti-inflammatory phenotype, alleviating inflammation. SCFAs also promote Treg differentiation while suppressing pro-inflammatory Th1 and Th17 cell function [111]. Tregs protect bone tissue by inhibiting osteoclastogenesis through cell-contact-dependent mechanisms and by secreting IL-10 and TGF-β to suppress osteoclast differentiation. Furthermore, Tregs release amphiregulin, a growth factor that stimulates MuSC activity and promotes muscle regeneration.

A balanced microbiota preserves intestinal barrier integrity by modulating host immunity and producing SCFAs. Conversely, dysbiosis disrupts this barrier, facilitating microbial translocation and triggering systemic inflammation, which contributes to concurrent muscle and bone loss in patients with OS. Among SCFAs, butyrate, propionate, and acetate bolster the epithelial barrier and orchestrate mucosal immune responses. As the primary metabolic substrate for colonocytes, butyrate promotes epithelial proliferation and differentiation, thereby sustaining barrier robustness. Moreover, butyrate stimulates mucin secretion to reinforce epithelial defense and inhibit pathogen adhesion. By upregulating the expression of tight junction proteins, SCFAs reduce intestinal permeability, limiting bacterial translocation and subsequent leak-associated inflammation [112].

In summary, immune cells form a “metabolic-immune” regulatory node through subpopulation differentiation and polarization. Interactions between immune cells and metabolic pathways exhibit tissue specificity, with potentially distinct mechanisms in skeletal muscle versus bone. Elucidating how immune cells modulate the local metabolic microenvironment may provide theoretical support for novel therapeutic strategies.

## 7. Multi-Omics Approaches for Biomarker Discovery and Patient Stratification

Given the heterogeneity of OS, single biomarkers are unlikely to capture disease complexity. Multi-omics approaches offer the opportunity to define molecular subtypes, identify early biomarkers, and guide individualised interventions. Metabolomics can detect alterations in amino acids, lipids, bile acids, microbial metabolites, acylcarnitines, and energy-related intermediates, thereby linking systemic metabolism to muscle and bone phenotypes. Proteomics can identify inflammatory mediators, extracellular matrix proteins, myokines, osteokines, complement factors, and senescence-associated proteins that reflect tissue remodelling and immune activation. Transcriptomics, including single-cell RNA sequencing, can reveal cell-specific changes in osteoblasts, osteoclasts, osteocytes, satellite cells, immune cells, and mesenchymal progenitors. Epigenomics may clarify how ageing, sex hormones, inflammation, diet, and exercise reprogramme musculoskeletal and immune cell function.

Spatial omics is particularly promising because OS involves tissue-level interactions among bone marrow, muscle, adipose tissue, vasculature, and immune niches. Spatial transcriptomics and imaging mass cytometry may help identify local immune–metabolic niches, such as inflammatory bone marrow microenvironments or impaired muscle-regenerative zones. Integrating multi-omics data with clinical phenotypes, imaging, physical performance, dietary patterns, medication exposure, and microbiome profiles could enable patient stratification into inflammation-dominant, mitochondrial dysfunction-dominant, adiposity-dominant, gut dysbiosis-dominant, or hormone-related subtypes. Such stratification would be especially valuable for clinical trials, in which biologically selected patients may respond more clearly to targeted immunometabolic interventions.

## 8. Sex-Specific Biology in OS

Sex-specific biology is clinically important in OS because women and men differ in hormonal regulation, body composition, immune responses, mitochondrial function, and patterns of musculoskeletal ageing. In women, menopause-related oestrogen deficiency accelerates bone resorption, increases pro-inflammatory cytokine production, alters adipose distribution, and impairs mitochondrial function. Oestrogen also modulates osteocyte viability, osteoblast activity, osteoclast differentiation, satellite cell function, and immune cell polarisation. These mechanisms may partly explain the high prevalence of osteopenia and OP in older women and the frequent coexistence of low muscle strength after menopause.

In men, gradual testosterone decline, altered androgen receptor signalling, increased visceral adiposity, and metabolic syndrome may contribute to muscle loss, insulin resistance, and impaired bone quality. Testosterone supports muscle protein synthesis, neuromuscular function, and bone formation, whereas low testosterone is associated with frailty and reduced physical performance. Sex differences in immune ageing are also relevant: women generally mount stronger immune responses but may be more susceptible to certain inflammatory or autoimmune phenotypes, whereas men may exhibit distinct patterns of immunosenescence and metabolic inflammation. Future studies should report sex-stratified results and examine whether diagnostic thresholds, biomarkers, and interventions require sex-specific optimisation.

## 9. Therapeutic Implications

OS arises from the synergistic detrimental effects of immune dysregulation and systemic metabolic disorders on the musculoskeletal system. At present, physical activity and nutritional optimization have the strongest clinical support for preserving muscle function, reducing falls, and improving bone-related outcomes. By contrast, many immunometabolic therapies, including senolytics, NAD+-boosting compounds, mitochondrial antioxidants, AMPK activators, mTOR modulators, and microbiome-directed interventions, are supported mainly by animal studies, small clinical studies, or mechanistic evidence. While recent advancements in multi-omics and clinical research have begun to fill the theoretical gaps in our understanding of the OS immunometabolic landscape, translating these mechanistic insights into effective clinical interventions remains a primary objective.

Currently, the clinical management of OS relies on a combination of lifestyle modifications and bone-specific pharmacological agents. Non-pharmacological strategies, primarily exercise-based interventions and nutritional support, are considered the cornerstone for managing musculoskeletal decline. Exercise improves muscle function and bone density [113]. Resistance training stimulates muscle protein synthesis through mTORC1 activation, improves neuromuscular recruitment and increases muscle strength [114]. Aerobic exercise improves mitochondrial biogenesis through AMPK-SIRT1-PGC-1alpha signaling, and lowers systemic inflammation [115]. It is particularly important to develop an appropriate exercise programme that combines resistance training and aerobic exercise, as this can simultaneously address muscle strength, bone loading, gait stability and the risk of falls. Exercise also modulates the mechanisms discussed in this review. It reduces chronic low-grade inflammation, improves macrophage polarization toward pro-resolution phenotypes, enhances Treg function, suppresses excessive oxidative stress, stimulates mitochondrial turnover, improves glucose and lipid metabolism, and alters gut microbiota composition and microbial metabolite production. Although exercise protocols vary across studies, progressive resistance training combined with adequate protein and vitamin D intake is currently the most clinically actionable strategy. Future trials should determine whether exercise responsiveness differs across immunometabolic subtypes of OS. Nutritional interventions (protein, vitamin D, antioxidants) enhance muscle mass and bone metabolism [116].

**Figure 2:**
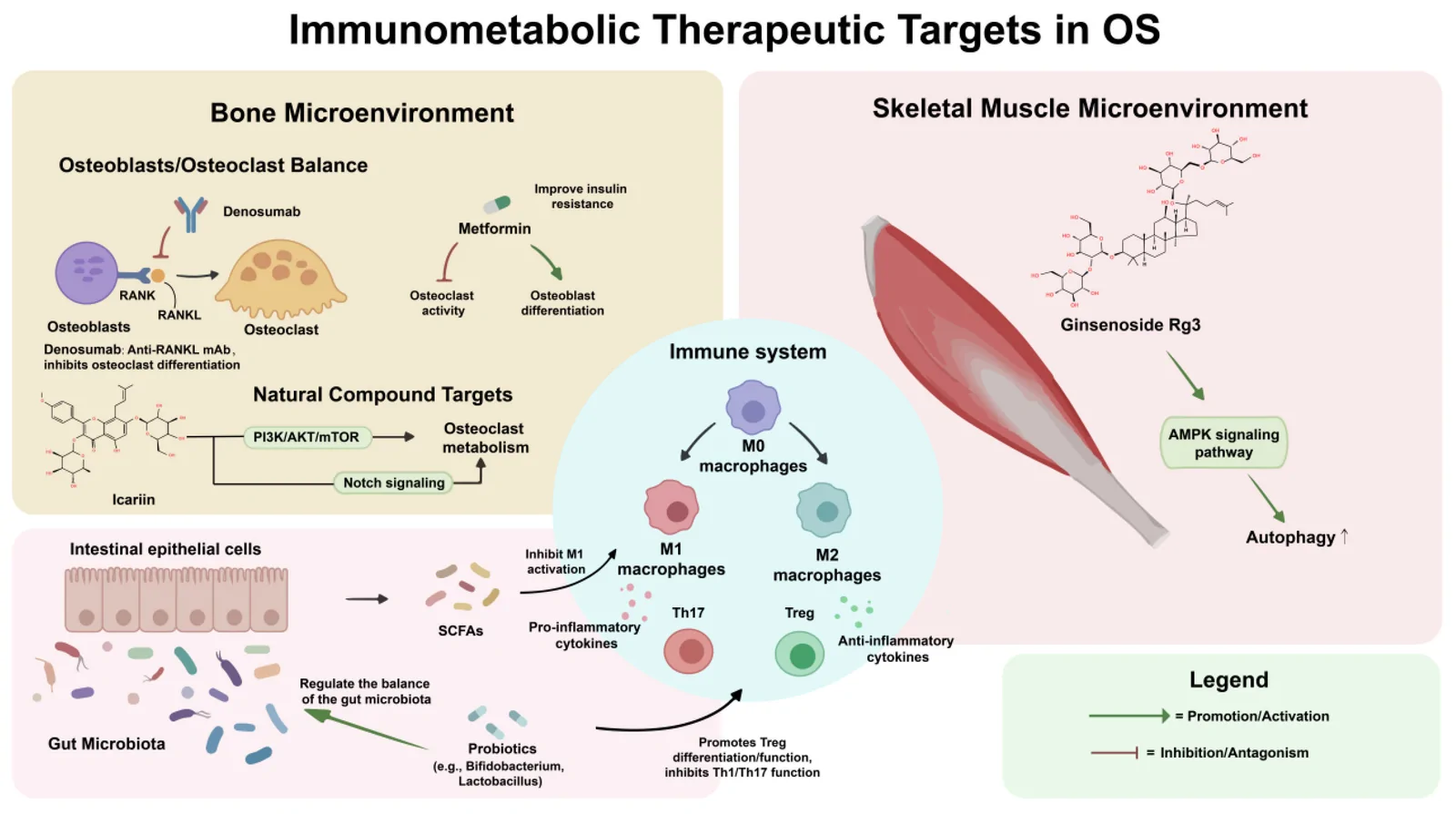
The effects of different drugs on OS.

In terms of pharmacotherapy, no specific drugs exist for SP. Conventional OP treatments—denosumab, romosozumab, bisphosphonates—target bone resorption. Denosumab, an anti-RANKL monoclonal antibody, inhibits osteoclast differentiation by suppressing NF-κB signaling. However, these drugs fail to address underlying metabolic inflammation driving OS. Anti-inflammatory biologics—TNF-α inhibitors (etanercept) or IL-6 receptor antagonists (tocilizumab)—represent potential strategies to mitigate inflammaging in OS patients. Targeted therapies, gene therapies, and stem cell therapies offer additional avenues, though further research is needed to elucidate mechanisms. Figure 2 illustrates the effects of different drugs on OS.

Emerging therapies aim to target upstream biological aging and metabolic dysfunction. Senolytics may reduce senescent-cell burden and SASP signaling, potentially improving bone remodeling and muscle regeneration, but clinical evidence in OS is lacking. NAD+-boosting therapies, including nicotinamide riboside and nicotinamide mononucleotide, may enhance mitochondrial function and sirtuin signaling; however, musculoskeletal benefits remain uncertain. AMPK activators such as metformin may improve insulin sensitivity, inflammation, and mitochondrial homeostasis, but their effects on muscle mass and bone outcomes may depend on age, dose, exercise status, and metabolic phenotype. mTOR modulation is biologically complex: mTORC1 activation supports muscle protein synthesis, whereas chronic overactivation may impair autophagy and promote aging-related dysfunction. Therefore, timing, tissue specificity, and patient selection are critical.

Mitochondrial antioxidants, including mitochondria-targeted compounds, may reduce oxidative damage and improve mitochondrial resilience in preclinical models. GLP-1 receptor agonists and sodium-glucose cotransporter 2 inhibitors may indirectly influence OS through weight reduction, improved glycemic control, lower inflammation, and altered substrate metabolism, but potential effects on lean mass, bone turnover, falls, and fracture risk require careful evaluation. Future therapies may focus on modulating immune subsets, such as enhancing Treg function or suppressing Th17 differentiation, to restore musculoskeletal equilibrium.

## 10. Challenges and Future Perspectives

Despite significant advances in mechanistic research, numerous challenges persist. First, existing studies predominantly rely on animal models, which often fail to adequately simulate the complex human immunometabolic network or replicate disease states induced by long-term lifestyle factors, potentially introducing experimental bias. Furthermore, the pathogenesis of OS is influenced by an intricate interplay of genetic, environmental, and lifestyle factors, resulting in marked patient heterogeneity. Future research must prioritize advancing personalized medicine by employing multidimensional analyses to integrate individual genetic backgrounds with environmental exposure data, thereby optimizing non-pharmacological and targeted therapeutic strategies. It is also essential to integrate metabolomics technologies to screen relevant biomarkers for early detection and treatment response monitoring. Additionally, deepening investigations into the precise molecular dialogue between immune cells and metabolic dysfunction will facilitate the development of highly specific inhibitors or agonists targeting metabolic enzymes. In summary, a comprehensive therapeutic\strategy—fusing anti-inflammatory, metabolic regulation, and tissue protection functions—is crucial for the early diagnosis and holistic management of OS.

## Figures and Tables

**Table 1:** Dual Effects of Key Cytokines on Bone and Muscle Systems in OS.

Factor	Characterization	Effects on Bone	Effects on Muscle	Key Molecular Mechanisms
IL-6	Pro-inflammatory	Stimulates RANKL expression	Promoting osteoclast differentiation and bone resorption	Induces skeletal muscle protein breakdown and atrophy
TNF-α	Pro-inflammatory	Promotes osteoclast differentiation and bone resorption via RANKL stimulation	Impairs myogenesis and stimulates protein degradation via the ubiquitin-proteasome system	Inhibits the Akt/mTOR signaling pathway
IL-17	Pro-inflammatory	Promotes osteoclast differentiation and mediates bone loss	Contributes to the inflammatory milieu driving musculoskeletal decline	Acts via IL-17RA and adaptor protein Act1
IL-1β	Pro-inflammatory	Impairs osteogenic differentiation of mesenchymal stem cells (MSCs)	Contributes to systemic inflammation and muscle catabolism	Stabilization of HIF-1α under hypoxic/metabolic stress
IL-10	Anti-inflammatory	Suppresses osteoclast generation and differentiation	Protecting bone mass	Mitigates inflammation and promotes muscle fiber regeneration
TGF-β	Anti-inflammatory	Regulates osteoprogenitor cell differentiation and bone homeostasis	Promotes MuSC proliferation and facilitates myocyte regeneration	Activation of the Smad signaling pathway

## Data Availability

No new data were created or analyzed in this study. Data sharing is not applicable to this article.

## Abbreviation

OS	Osteosarcopenia
EWGSOP2	the European Working Group on Sarcopenia in Older People 2
AWGS	the Asian Working Group for Sarcopenia
IOF	the International Osteoporosis Foundation
ESCEO	European Society for Clinical and Economic Aspects of Osteoporosis, Osteoarthritis and Musculoskeletal Diseases
BIA	Bioelectrical impedance analysis
DXA	Dual-energy X-ray absorptiometry
CT	Computed tomography
BMD	Bone mineral density
IGF-1	Insulin-like growth factor 1
IL	Interleukin
SASP	Senescence-associated secretory phenotype
TNF-α	Tumour necrosis factor-α
TGF-β	Transforming growth factor-β
RANKL	Receptor Activator of Nuclear Factor-κB Ligand
JAK	Janus activated kinase
STAT	Signal transducer and activator of transcription
SOCS	Suppressor of cytokine signaling
IRS1	Insulin receptor substrate-1
Akt	Protein Kinase B
mTOR	Mammalian target of rapamycin
Tregs	Regulatory T cells
IL-17RA	IL-17 receptor A
Act1	Nuclear Factor Kappa-B activator 1
IFN-γ	Interferon-gamma
HIF-1α	Hypoxia-inducible factor-1α
FAO	Fatty acid oxidation
OXPHOS	oxidative phosphorylation
OP	Osteoporosis
OPG	Osteoprotegerin
Bregs	Regulatory B cells
PPP	Pentose phosphate pathway
TCA	Tricarboxylic acid
PDK1	Pyruvate dehydrogenase kinase 1
AMPK	AMP-activated protein kinase
VEGF	Vascular Endothelial Growth Factor
Runx2	Runt-related transcription factor 2
ROS	Reactive oxygen species
SP	Sarcopenia
mtDNA	mitochondrial DNA
MuSC	muscle stem cell
ATG7	autophagy-related gene 7
AGEs	Advanced glycation end-products
BMSC	Bone marrow mesenchymal stem cell
Sirt3	Sirtuin 3
UPRmt	the mitochondrial unfolded protein response
MDPs	Mitochondrial-derived peptides
PGC-1α	Peroxisome proliferator-activated receptor-γ coactivator-1α
NRF1/2	Nuclear respiratory factors
SCFAs	Short-chain fatty acids
HDAC	Histone deacetylase
FXR	Farnesoid X receptor
BSH	Bile salt hydrolase
FGF15	Fibroblast growth factor 15
FGFR4/KLB	Fibroblast growth factor receptor 4/β-klotho
T2DM	Type 2 diabetes mellitus
RAGE	Receptor for Advanced Glycosylation End-Products
TyG index	Triglyceride-glucose index
AIP	Atherogenic index
FFAs	Free fatty acids
MSCs	Mesenchymal stem cells
SDH	Succinate dehydrogenase
ERK1/2	Extracellular signal-regulated kinase 1/2
DRP1	Dynamin-related protein 1
FFARs	Free fatty acid receptors
BCAAs	Branched-chain amino acids
mTORC1	mTOR complex 1
BCKAs	Branched-chain α-keto acids
SAM	S-adenosylmethionine
SHMT	Serine hydroxymethyltransferase
AhR	Aryl hydrocarbon receptor
NF-κB	Nuclear factor-κB
TMAO	Trimethylamine N-oxide

## References

[1] Veronese N., Ragusa F.S., Sabico S., Dominguez L.J., Barbagallo M., Duque G., Al-Daghri N. (2024). Osteosarcopenia increases the risk of mortality: A systematic review and meta-analysis of prospective observational studies. Aging Clin. Exp. Res..

[2] Solla-Suarez P., Arif S.G., Ahmad F., Rastogi N., Meng A., Cohen J.M., Rodighiero J., Piazza N., Martucci G., Lauck S. (2024). Osteosarcopenia and mortality in older adults undergoing transcatheter aortic valve replacement. JAMA Cardiol..

[3] Liu Y.D., Deng Q., Zhang Y.J., Li Z.F., Peng R.D., Guo T.F., Wang Y.R., Chen B. (2024). Research progress on signaling pathways related to musculoskeletal symbiosis. Peking Union Med. J..

[4] Zhang X.Y., Wu D., Zhao E.Z., Song X.B., Zhang X.L. (2025). Treatment and repair of musculoskeletal degenerative diseases and injuries from the perspective of musculoskeletal interaction mechanisms. Chin. J. Tissue Eng. Res..

[5] Mogilenko D.A., Sergushichev A., Artyomov M.N. (2023). Systems immunology approaches to metabolism. Annu. Rev. Immunol..

[6] Marmondi F., Ferrando V., Filipas L., Codella R., Ruggeri P., La Torre A., Faelli E.L., Bonato M. (2025). Exercise-induced biomarker modulation in sarcopenia: From inflamm-aging to muscle regeneration. Sports.

[7] Huang J.C., Huang H.X., Wan L., Lin Y.P. (2021). Analysis of the spleen-kidney-muscle-bone-mitochondria theory. Chin. J. Osteoporos..

[8] Lu C., Wang H., Li C., Lyu H., Shen L., Zhang J., Rong W., Wei J., Zeng C., Lei G. (2025). Disease-associated and shared gut microbes of sarcopenia and osteoporosis: A systematic review and meta-analysis. Aging Dis..

[9] Cruz-Jentoft A.J., Bahat G., Bauer J., Boirie Y., Bruyère O., Cederholm T., Cooper C., Landi F., Rolland Y., Sayer A.A. (2019). Sarcopenia: Revised European consensus on definition and diagnosis. Age Ageing.

[10] Jianghan B., Jiaojiao L., Lin K. (2026). Interpretation of A Focus Shift from Sarcopenia to Muscle Health in the Asian Working Group for Sarcopenia 2025 Consensus Update. Aging Med..

[11] Shevroja E., Reginster J.Y., Lamy O., Al-Daghri N., Chandran M., Demoux-Baiada A.L., Kohlmeier L., Lecart M.P., Messina D., Camargos B.M. (2023). Update on the clinical use of trabecular bone score (TBS) in the management of osteoporosis: Results of an expert group meeting organized by the European Society for Clinical and Economic Aspects of Osteoporosis, Osteoarthritis and Musculoskeletal Diseases (ESCEO), and the International Osteoporosis Foundation (IOF) under the auspices of WHO Collaborating Center for Epidemiology of Musculoskeletal Health and Aging. Osteoporos. Int..

[12] Liu Y.D., Deng Q., Zhang Y.J., Li Z.F., Peng R.D., Chen B. (2024). Research progress on the role of regulatory T cell/Th17 cell balance in musculoskeletal attenuation and reconstruction. Chin. J. Cell. Mol. Immunol..

[13] Zhang S., Ni W. (2023). High systemic immune-inflammation index is relevant to osteoporosis among middle-aged and older people: A cross-sectional study. Immun. Inflamm. Dis..

[14] Wang B., Han J., Elisseeff J.H., Demaria M. (2024). The senescence-associated secretory phenotype and its physiological and pathological implications. Nat. Rev. Mol. Cell Biol..

[15] Salamanna F., Faldini C., Veronesi F., Borsari V., Ruffilli A., Manzetti M., Viroli G., Traversari M., Marchese L., Fini M. (2024). A pilot study on circulating, cellular, and tissue biomarkers in osteosarcopenic patients. Int. J. Mol. Sci..

[16] Yan L., Hu R., Tu S., Cheng W.J., Zheng Q., Wang J.W., Kan W.S., Ren Y.J. (2015). Meta-analysis of association between IL-6-634C/G polymorphism and osteoporosis. Genet. Mol. Res..

[17] Belizário J.E., Fontes-Oliveira C.C., Borges J.P., Kashiabara J.A., Vannier E. (2016). Skeletal muscle wasting and renewal: A pivotal role of myokine IL-6. SpringerPlus.

[18] Li J., Yi X., Yao Z., Chakkalakal J.V., Xing L., Boyce B.F. (2020). TNF receptor-associated factor 6 mediates TNFα-induced skeletal muscle atrophy in mice during aging. J. Bone Min. Res..

[19] Goodman M.N. (1991). Tumor necrosis factor induces skeletal muscle protein breakdown in rats. Am. J. Physiol..

[20] Haddad F., Zaldivar F., Cooper D.M., Adams G.R. (2005). IL-6-induced skeletal muscle atrophy. J. Appl. Physiol..

[21] Walston J., Fedarko N., Yang H., Leng S., Beamer B., Espinoza S., Lipton A., Zheng H., Becker K. (2008). The physical and biological characterization of a frail mouse model. J. Gerontol. A Biol. Sci. Med. Sci..

[22] Qi Z., Luo J., Xiao Z., Yang D. (2025). Functional roles of immune cells in osteoporosis. Front. Immunol..

[23] Fu Y., Wang B., Alu A., Hong W., Lei H., He X., Shi H., Cheng P., Yang X. (2025). Immunosenescence: Signaling pathways, diseases and therapeutic targets. Signal Transduct. Target. Ther..

[24] Liu Z., Liang Q., Ren Y., Guo C., Ge X., Wang L., Cheng Q., Luo P., Zhang Y., Han X. (2023). Immunosenescence: Molecular mechanisms and diseases. Signal Transduct. Target. Ther..

[25] Adamopoulos I.E., Chao C.C., Geissler R., Laface D., Blumenschein W., Iwakura Y., McClanahan T., Bowman E.P. (2010). Interleukin-17a upregulates receptor activator of NF-κB on osteoclast precursors. Arthritis Res. Ther..

[26] DeSelm C.J., Takahata Y., Warren J., Chappel J.C., Khan T., Li X., Liu C., Choi Y., Kim Y.F., Zou W. (2012). IL-17 mediates estrogen-deficient osteoporosis in an Act1-dependent manner. J. Cell. Biochem..

[27] Su J., Li Y., Zhang L., Chen X., Liu J., Jiang Z., Luo Y.Z., Tan S.H. (2024). Research progress on metabolic reprogramming and immune resistance of tumor infiltrating lymphocytes. Chin. J. Clin. Oncol..

[28] Schiaffino S., Pereira M.G., Ciciliot S., Rovere-Querini P. (2017). Regulatory T cells and skeletal muscle regeneration. FEBS J..

[29] Frase D., Lee C., Nachiappan C., Gupta R., Akkouch A. (2023). The inflammatory contribution of B-lymphocytes and neutrophils in progression to osteoporosis. Cells.

[30] Li Y., Toraldo G., Li A., Yang X., Zhang H., Qian W.P., Weitzmann M.N. (2007). B-lymphocytes and T-cells are critical for the preservation of bone homeostasis and attainment of peak bone mass in vivo. Blood.

[31] Rajakumar S.A., Papp E., Lee K.K., Grandal I., Merico D., Liu C.C., Allo B., Zhang L., Grynpas M.D., Minden M.D. (2020). B cell acute lymphoblastic leukemia cells mediate RANK-RANKL-dependent bone destruction. Sci. Transl. Med..

[32] Wang R.X., Yu C.R., Dambuza I.M., Mahdi R.M., Dolinska M.B., Sergeev Y.V., Wingfield P.T., Kim S.H., Egwuagu C.E. (2014). Interleukin 35 induces regulatory B cells that suppress autoimmune disease. Nat. Med..

[33] Johnstone J.C., Yazicioglu Y.F., Clarke A.J. (2024). Fuelling B cells: Dynamic regulation of B cell metabolism. Curr. Opin. Immunol..

[34] Waters L.R., Ahsan F.M., Wolf D.M., Shirihai O., Teitell M.A. (2018). Initial B cell activation induces metabolic reprogramming and mitochondrial remodeling. iScience.

[35] Sheu K.M., Hoffmann A. (2022). Functional hallmarks of healthy macrophage responses: Their regulatory basis and disease relevance. Annu. Rev. Immunol..

[36] Zhu L., Zhao Q., Yang T., Ding W., Zhao Y. (2015). Cellular metabolism and macrophage functional polarization. Int. Rev. Immunol..

[37] Min B.K., Park S., Kang H.J., Kim D.W., Ham H.J., Ha C.M., Choi B.J., Lee J.Y., Oh C.J., Yoo E.K. (2019). Pyruvate dehydrogenase kinase is a metabolic checkpoint for polarization of macrophages to the M1 phenotype. Front. Immunol..

[38] Shimada N., Sakata A., Igarashi T., Takeuchi M., Nishimura S. (2020). M1 macrophage infiltration exacerbates muscle/bone atrophy after peripheral nerve injury. BMC Musculoskelet. Disord..

[39] Deng L., Jian Z., Xu T., Li F., Deng H., Zhou Y., Lai S., Xu Z., Zhu L. (2023). Macrophage polarization: An important candidate regulator for lung diseases. Molecules.

[40] Dussold C., Zilinger K., Turunen J., Heimberger A.B., Miska J. (2024). Modulation of macrophage metabolism as an emerging immunotherapy strategy for cancer. J. Clin. Investing..

[41] Li X., Kong L.J., Deng Y.L., Zhang J.L., Sun X.X., Han S.L., Meng H.J., Wang Z.S. (2025). Research progress on the mechanism of macrophage polarization in musculoskeletal degenerative diseases. Chin. J. Osteoporos..

[42] Muñoz J., Akhavan N.S., Mullins A.P., Arjmandi B.H. (2020). Macrophage polarization and osteoporosis: A review. Nutrients.

[43] Mahon O.R., Browe D.C., Gonzalez-Fernandez T., Pitacco P., Whelan I.T., Von Euw S., Hobbs C., Nicolosi V., Cunningham K.T., Mills K.H.G. (2020). Nanoparticle mediated M2 macrophage polarization enhances bone formation and MSC osteogenesis in an IL-10 dependent manner. Biomaterials.

[44] Zeng Y.J., Li X.Y., Chen T.Y., Liu S.H., Cai Q.Z., Yang Y.F., Yang D.S., Lei S.F., Liu B., Wan L. (2026). Relationship between senile osteoporosis and mitochondrial dysfunction and therapeutic strategies of traditional Chinese medicine. J. Tradit. Chin. Orthop. Traumatol..

[45] Gao X., Yu X., Zhang C., Wang Y., Sun Y., Sun H., Zhang H., Shi Y., He X. (2022). Telomeres and mitochondrial metabolism: Implications for cellular senescence and age-related diseases. Stem Cell Rev. Rep..

[46] Dobson P.F., Dennis E.P., Hipps D., Reeve A., Laude A., Bradshaw C., Stamp C., Smith A., Deehan D.J., Turnbull D.M. (2020). Mitochondrial dysfunction impairs osteogenesis, increases osteoclast activity, and accelerates age-related bone loss. Sci. Rep..

[47] Pignolo R.J., Chandra A. (2025). Insights into age-related osteoporosis from senescence-based preclinical models and human accelerated aging paradigms. Mech. Ageing Dev..

[48] Liu J., Gao Z., Liu X. (2024). Mitochondrial dysfunction and therapeutic perspectives in osteoporosis. Front. Endocrinol..

[49] Dong X., Wang X.Q., Wang X.H., Mo Y.H. (2022). Research progress on the role of oxidative stress in the pathogenesis of sarcopenia. Chin. J. Gerontol..

[50] Chen K., Qiu P., Yuan Y., Zheng L., He J., Wang C., Guo Q., Kenny J., Liu Q., Zhao J. (2019). Pseudorotin inhibits osteoclastogenesis and prevents ovariectomized-induced bone loss by suppressing reactive oxygen species. Theranostics.

[51] Feng X., Liu Z., Su Y., Lian H., Gao Y., Zhao J., Xu J., Liu Q., Song F. (2023). Tussilagone inhibits osteoclastogenesis by modulating mitochondrial function and ROS production involved Nrf-2 activation. Biochem. Pharmacol..

[52] Martinez-Vicente M. (2017). Neuronal mitophagy in neurodegenerative diseases. Front. Mol. Neurosci..

[53] Su S.H., Wu Y.F., Wang D.P., Hai J. (2018). Inhibition of excessive autophagy and mitophagy mediates neuroprotective effects of URB597 against chronic cerebral hypoperfusion. Cell Death Dis..

[54] Cairns G., Thumiah-Mootoo M., Abbasi M.R., Gourlay M., Racine J., Larionov N., Prola A., Khacho M., Burelle Y. (2024). PINK1 deficiency alters muscle stem cell fate decision and muscle regenerative capacity. Stem Cell Rep..

[55] Carnio S., LoVerso F., Baraibar M.A., Longa E., Khan M.M., Maffei M., Reischl M., Canepari M., Loefler S., Kern H. (2014). Autophagy impairment in muscle induces neuromuscular junction degeneration and precocious aging. Cell Rep..

[56] El Assar M., Álvarez-Bustos A., Sosa P., Angulo J., Rodríguez-Mañas L. (2022). Effect of Physical Activity/Exercise on Oxidative Stress and Inflammation in Muscle and Vascular Aging. Int. J. Mol. Sci..

[57] Alizadeh Pahlavani H., Laher I., Knechtle B., Zouhal H. (2022). Exercise and mitochondrial mechanisms in patients with sarcopenia. Front. Physiol..

[58] Yu B., Huo L., Liu Y., Deng P., Szymanski J., Li J., Luo X., Hong C., Lin J., Wang C.Y. (2018). PGC-1α Controls Skeletal Stem Cell Fate and Bone-Fat Balance in Osteoporosis and Skeletal Aging by Inducing TAZ. Cell Stem Cell.

[59] Zhu Y., Zhou X., Zhu A., Xiong S., Xie J., Bai Z. (2023). Advances in exercise to alleviate sarcopenia in older adults by improving mitochondrial dysfunction. Front. Physiol..

[60] Bai J., Zhang L., He L., Zhou Y. (2025). Altered mitochondrial function: A clue therapeutic strategies between metabolic dysfunction-associated steatotic liver disease and chronic kidney disease?. Front. Nutr..

[61] Wu Z., Dai Q., Wang Y., Wu N., Wang C., Shi J. (2025). Emerging roles of the metabolite succinate in bone-related diseases. J. Zhejiang Univ. Sci. B..

[62] Torres A.K., Fleischhart V., Inestrosa N.C. (2024). Mitochondrial unfolded protein response (UPR^mt^): What we know thus far. Front. Cell Dev. Biol..

[63] Wang Y., Li J., Zhang Z., Wang R., Bo H., Zhang Y. (2023). Exercise Improves the Coordination of the Mitochondrial Unfolded Protein Response and Mitophagy in Aging Skeletal Muscle. Life.

[64] Zhu S., Hu X., Bennett S., Xu J., Mai Y. (2022). The Molecular Structure and Role of Humanin in Neural and Skeletal Diseases, and in Tissue Regeneration. Front. Cell Dev. Biol..

[65] Kumagai H., Coelho A.R., Wan J., Mehta H.H., Yen K., Huang A., Zempo H., Fuku N., Maeda S., Oliveira P.J. (2021). MOTS-c reduces myostatin and muscle atrophy signaling. Am. J. Physiol. Endocrinol. Metab..

[66] Xu Z.K., Gao Y. (2025). Research progress on abnormal glucose and lipid metabolism and pathogenesis of sarcopenia. Chin. J. Diabetes.

[67] Sun X.L., Xia T.S., Zhang S.Y., Zhang J.B., Xu L.C., Han T., Xin H.L. (2022). Hops extract and xanthohumol ameliorate bone loss induced by iron overload via activating Akt/GSK3β/Nrf2 pathway. J. Bone Min. Metab..

[68] Zhu H.W., Shi D.J., Li S.Z. (2022). Genistein inhibits osteoarthritis rabbit chondrocyte degeneration by activating PI3K/AKT/mTOR signaling pathway. Med. J. Wuhan Univ..

[69] Zhou B., Chen Y.C., Qiu L.L., Wang Y.L., Zhang L.Z., Li X.Y. (2026). Influencing factors of type 2 diabetes complicated with osteoporosis and correlation of serum AKT, GSK-3β levels with bone mineral density. Med. J. Wuhan Univ..

[70] Du H., Ma Y., Wang X., Zhang Y., Zhu L., Shi S., Pan S., Liu Z. (2023). Advanced glycation end products induce skeletal muscle atrophy and insulin resistance via activating ROS-mediated ER stress PERK/FOXO1 signaling. Am. J. Physiol. Endocrinol. Metab..

[71] Wu D., Cline-Smith A., Shashkova E., Perla A., Katyal A., Aurora R. (2021). T-cell mediated inflammation in postmenopausal osteoporosis. Front. Immunol..

[72] Khalid M., Petroianu G., Adem A. (2022). Advanced glycation end products and diabetes mellitus: Mechanisms and perspectives. Biomolecules.

[73] Aung M., Amin S., Gulraiz A., Gandhi F.R., Pena Escobar J.A., Malik B.H. (2020). The future of metformin in the prevention of diabetes-related osteoporosis. Cureus.

[74] Chen T.X., Dong T.T., Li Y., Zhang S., Zhang L. (2024). Mendelian randomization analysis of causal relationship between blood metabolites and sarcopenia-related traits. Chin. J. Tissue Eng. Res..

[75] Al Saedi A., Debruin D.A., Hayes A., Hamrick M. (2022). Lipid metabolism in sarcopenia. Bone.

[76] Li C.W., Yu K., Shyh-Chang N., Jiang Z., Liu T., Ma S., Luo L., Guang L., Liang K., Ma W. (2022). Pathogenesis of sarcopenia and the relationship with fat mass: Descriptive review. J. Cachexia Sarcopenia Muscle.

[77] Yao J., Xu J., Sun Y., Ji Y., Lou H., Wu X., Cong D. (2025). Association of triglyceride-glucose index and atherogenic index of plasma with sarcopenia across different glucose metabolism states: The mediating role of oxidative stress and chronic inflammation. Eur. J. Med. Res..

[78] Franco-Romero A., Sandri M., Schiaffino S. (2024). Autophagy in skeletal muscle. Cold Spring Harb. Perspect. Biol..

[79] Jiang T.X., Zou J.B., Zhu Q.Q., Liu C.H., Wang G.F., Du T.T., Luo Z.Y., Guo F., Zhou L.M., Liu J.J. (2019). SIP/CacyBP promotes autophagy by regulating levels of BRUCE/Apollon, which stimulates LC3-I degradation. Proc. Natl. Acad. Sci. USA.

[80] Zhao X.Y., Mo S.Q., Wang H.L., Cui S.H., Hua Y.Q. (2025). Research progress on the role of lipid catabolism and utilization in the pathological process of osteoporosis. Chin. J. Osteoporos..

[81] Gheller B.J., Blum J.E., Lim E.W., Handzlik M.K., Hannah Fong E.H., Ko A.C., Khanna S., Gheller M.E., Bender E.L., Alexander M.S. (2021). Extracellular serine and glycine are required for mouse and human skeletal muscle stem and progenitor cell function. Mol. Metab..

[82] Bras H., Liabeuf S. (2021). Differential effects of spinal cord transection on glycinergic and GABAergic synaptic signaling in sub-lesional lumbar motoneurons. J. Chem. Neuroanat..

[83] Huh J.E., Choi J.Y., Shin Y.O., Park D.S., Kang J.W., Nam D., Choi D.Y., Lee J.D. (2014). Arginine enhances osteoblastogenesis and inhibits adipogenesis through the regulation of Wnt and NFATc signaling in human mesenchymal stem cells. Int. J. Mol. Sci..

[84] Xu S., Zhao G., Hanigan M.D., Cantalapiedra-Hijar G., Li M. (2025). Branched-chain amino acids in muscle growth: Mechanisms, physiological functions, and applications. J. Anim. Sci. Biotechnol..

[85] Mei J., Yang F.Y., Gong Q. (2026). Branched-chain amino acids and insulin resistance in type 2 diabetes: From metabolic dysregulation to therapeutic targets. Front. Endocrinol..

[86] Mattocks D.A., Mentch S.J., Shneyder J., Ables G.P., Sun D., Richie J.P., Locasale J.W., Nichenametla S.N. (2017). Short term methionine restriction increases hepatic global DNA methylation in adult but not young male C57BL/6J mice. Exp. Gerontol..

[87] Amorim T., Kumar N.G., David N.L., Dion W., Pagadala T., Doshi N.K., Zhu B., Parkhitko A., Steinhauser M.L., Fazeli P.K. (2024). Methionine as a regulator of bone remodeling with fasting. JCI Insight.

[88] Holeček M. (2022). Serine Metabolism in Health and Disease and as a Conditionally Essential Amino Acid. Nutrients.

[89] Takatoya M., Kasugai T., Arai D., Kasuga U., Miyaura C., Hirata M., Itoh Y., Tominari T., Aoki Y., Inada M. (2025). Glutamine Promotes Myogenesis in Myoblasts Through Glutaminolysis-Mediated Histone H3 Acetylation That Enhances Myogenin Transcription. Nutrients.

[90] Yu Y., Newman H., Shen L., Sharma D., Hu G., Mirando A.J., Zhang H., Knudsen E., Zhang G.F., Hilton M.J. (2019). Glutamine Metabolism Regulates Proliferation and Lineage Allocation in Skeletal Stem Cells. Cell Metab..

[91] Chen M., Wang J., Yang Y., He Y., Li L. (2025). The interplay of estrogen, gut microbiome, and bone immunity in osteoporosis. Cell Commun. Signal..

[92] Hu W., Shi J., Lu Y., Wu H., Li G., Yin X., Jiao D., Li J., Wu L., Zhao C. (2025). Moxibustion modulates gut microbiota and improves bone marrow hematopoiesis in mice with myelosuppression-induced aplastic. World. J. Acupunct. Moxibustion.

[93] Kimura I., Ichimura A., Ohue-Kitano R., Igarashi M. (2020). Free fatty acid receptors in health and disease. Physiol. Rev..

[94] Ahmad S., Ali S., Ur Rehman A., Ul Haq I., Liaqat I., Aftab M.N., Shume T. (2026). SCFAs’ pleiotropic role in pathogenesis and salutogenesis: Mechanisms in exacerbation and regulation of inflammation and fibrosis from gut to host. J. Med. Microbiol..

[95] Wei W., Wang S.X., Zhan L.B. (2024). Study on the correlation between osteosarcopenia and gut microbiota. World Sci. Technol. Mod. Tradit. Chin. Med..

[96] Lucas S., Omata Y., Hofmann J., Böttcher M., Iljazovic A., Sarter K., Albrecht O., Schulz O., Krishnacoumar B., Krönke G. (2018). Short-chain fatty acids regulate systemic bone mass and protect from pathological bone loss. Nat. Commun..

[97] Björkegren J.L.M., Lusis A.J. (2022). Atherosclerosis: Recent developments. Cell.

[98] Wahlström A., Sayin S.I., Marschall H.U., Bäckhed F. (2016). Intestinal crosstalk between bile acids and microbiota and its impact on host metabolism. Cell Metab..

[99] Mancin L., Wu G.D., Paoli A. (2023). Gut microbiota-bile acid-skeletal muscle axis. Trends Microbiol..

[100] Sayin S.I., Wahlström A., Felin J., Jäntti S., Marschall H.U., Bamberg K., Angelin B., Hyötyläinen T., Orešič M., Bäckhed F. (2013). Gut microbiota regulates bile acid metabolism by reducing the levels of tauro-beta-muricholic acid, a naturally occurring FXR antagonist. Cell Metab..

[101] Qiu Y., Yu J., Ji X., Yu H., Xue M., Zhang F., Li Y., Bao Z. (2022). Ileal FXR-FGF15/19 signaling activation improves skeletal muscle loss in aged mice. Mech. Ageing Dev..

[102] Guo A., Li K., Tian H.C., Fan Z., Chen Q.N., Yang Y.F., Yu J., Wu Y.X., Xiao Q. (2021). FGF19 protects skeletal muscle against obesity-induced muscle atrophy, metabolic derangement and abnormal irisin levels via the AMPK/SIRT-1/PGC-α pathway. J. Cell Mol. Med..

[103] Wu L., Wu S., Deng Y., Li J., Zhang C., Wang Y., Zhao C., Gao L. (2026). Indole-3-aldehyde Preserves Gingival Epithelial Barrier Structure and Function Via AhR/Nrf2 Signaling Pathway. Inflammation.

[104] Zhao Y., Wang C., Qiu F., Liu J., Xie Y., Lin Z., He J., Chen J. (2024). Trimethylamine-N-oxide promotes osteoclast differentiation and oxidative stress by activating NF-κB pathway. Aging.

[105] Liu S., D’Amico D., Shankland E., Bhayana S., Garcia J.M., Aebischer P., Rinsch C., Singh A., Marcinek D.J. (2022). Effect of Urolithin a Supplementation on Muscle Endurance and Mitochondrial Health in Older Adults: A Randomized Clinical Trial. JAMA Netw. Open.

[106] Attili L., Rossi M.N., Di Santo R., Duranti G., Ceci R., Cervelli M. (2026). Polyamines and autophagy as a dynamic regulatory network in skeletal muscle regeneration and aging. Mech. Ageing Dev..

[107] Hou X.X., Li X.Y., Chen W.Q., Zhu W.K., Guo H.D., Zhao L. (2025). The interactions between gut microbiota and Chinese herbal medicine in colorectal cancer. Acupunct. Herb. Med..

[108] Hor Y.Y., Ooi C.H., Lew L.C., Jaafar M.H., Lau A.S., Lee B.K., Azlan A., Choi S.B., Azzam G., Liong M.T. (2021). The molecular mechanisms of probiotic strains in improving ageing bone and muscle of D-galactose-induced ageing rats. J. Appl. Microbiol..

[109] Zhang S.H., Yang M.Y., Tang S.W., Wang C.Y., You J., Xie J., Chen X.H., Chen S.S. (2024). Effect of probiotics in the treatment of elderly patients with osteoporosis. Chin. Contemp. Med..

[110] Murphy M.P., O’Neill L.A.J. (2018). Krebs cycle reimagined: The emerging roles of succinate and itaconate as signal transducers. Cell.

[111] Furusawa Y., Obata Y., Fukuda S., Endo T.A., Nakato G., Takahashi D., Nakanishi Y., Uetake C., Kato K., Kato T. (2013). Commensal microbe-derived butyrate induces the differentiation of colonic regulatory T cells. Nature.

[112] Zhang Y., Pan J., Liu Y., Zhang X., Cheng K. (2023). Effects of *Ficus pandurata Hance* var. *angustifolia* Cheng flavonoids on intestinal barrier and cognitive function by regulating intestinal microbiota. Foods.

[113] Xie Y., Xu S., Dai R., Xu G., Bao H., Dong X., Xu Y., Ruan H., Chen L., Fu K. (2025). Comprehensive review of therapeutic efficacy and underlying mechanisms of various exercise modalities in treating osteoporosis. Aging Dis..

[114] Zhao Y.C. (2025). Dual roles of mTOR in skeletal muscle adaptation: Coordinating hypertrophic and mitochondrial biogenesis pathways for exercise-induced chronic disease management. Front. Med..

[115] Chen J., Liu B., Yao X., Yang X., Sun J., Yi J., Xue F., Zhang J., Shen Y., Chen B. (2025). AMPK/SIRT1/PGC-1α Signaling Pathway: Molecular Mechanisms and Targeted Strategies from Energy Homeostasis Regulation to Disease Therapy. CNS Neurosci. Ther..

[116] Kositsawat J., Duque G., Kirk B. (2021). Nutrients with anabolic/anticatabolic, antioxidant, and anti-inflammatory properties: Targeting the biological mechanisms of aging to support musculoskeletal health. Exp. Gerontol..

